# Effects of Regular Physical Exercises in the Water on the Metabolic Profile of Women with Abdominal Obesity

**DOI:** 10.2478/hukin-2014-0034

**Published:** 2014-07-08

**Authors:** Zbigniew Kasprzak, Łucja Pilaczyńska-Szcześniak

**Affiliations:** 1 Department of Hygiene, Chair of Physiology, Biochemistry and Hygiene, University School of Physical Education in Poznań, Poland.

**Keywords:** water aerobics, blood lipid profile, OGTT

## Abstract

Recreational physical exercise in the water is predominantly based on aerobic metabolism. Since it involves both carbohydrate and lipid sources of energy, aqua aerobics has a beneficial effect on metabolism of these substrates. The aim of the study was to assess the impact of a 3 month aqua aerobics training program on the metabolic profile of women with abdominal obesity. The study sample comprised 32 women aged 41–72 years. Somatic characteristics and variables characterizing carbohydrate and lipid metabolism were measured before the commencement and after the completion of the training program. During the 2nd measurement all mean anthropometric variables were found to be significantly lower (p≤0.01). In the blood lipid profile, the concentrations of total cholesterol, LDL-cholesterol and HOMAIR were significantly lower (p<0.01). Furthermore, the levels of fasting triglycerides, glucose and insulin were reduced significantly (p≤0.05) after the training program.

The aqua aerobics program contributed to positive changes in lipid metabolism, anthropometric variables, as well as the fasting insulin, glucose levels and insulin resistance index in women with abdominal obesity.

## Introduction

Abdominal obesity is commonly associated with hyperinsulinemia, insulin resistance and changes in the blood lipid profile. We recognize it, when waist circumference in women is at least 80 cm and the waist-hip ratio (WHR) is greater than 0,8. Researchers have indicated that a greater body mass is not only accompanied by insulin hypersecretion, but also lower hepatic clearance and by peripheral insulin resistance (Fujioka et al., 1987; Goodpaster et al., 2005). The age and lifestyle of the present-day population characterized by limited physical activity and a positive energy balance additionally facilitate metabolic disturbances (Chakravarthy et al., 2002; Karolkiewicz et al., 2009). At the same time, regular moderate physical activity, with a predominance of aerobic metabolism, is considered to be a major factor preventing obesity as well as resultant disease entities (Dylewicz et al., 2000). This is confirmed by numerous studies which found physical activity to increase insulin sensitivity, normalize the blood lipid profile and blood pressure in individuals with metabolic disorders (Gaziano et al., 1997; Dela et al., 1994).

One of the forms of regular physical activity often taken up by women includes aqua aerobics. This type of exercise is predominantly based on aerobic metabolic processes involving carbohydrate as well as lipid substrates (Romijn et al., 1993), and thus, improves carbohydrate and lipid metabolism.

The aim of the study was to evaluate the effects of a three-month recreational aqua aerobics program on the metabolic profile of women with abdominal obesity.

## Material and Methods

The study sample consisted of 32 women with abdominal obesity aged 41–72 years (52.80±6.63 years), who declared good health. The women volunteered to participate in aqua aerobics classes twice a week, for three months (having obtained a respective permission from their primary care physician). The subjects provided their written consent to participate in the tests, and the study was approved by the Bioethical Commission of the Poznań University of Medical Sciences. During the study the participants were advised not to change their dietary habits and lifestyle.

Two measurements of somatic traits (body mass, body height, waist circumference, hip circumference) and venous blood samples were taken before and after the training program, in the morning while fasting. Body mass and body height were measured using certified equipment Radwag (Radom, Poland) with an accuracy of 0.01 kg (body mass) and 0.5 cm (body height). Waist and hip circumferences were measured according to the WHO using measuring tape inextensible. Waist circumference was measured at half the distance between the bottom edge of the last rib and iliac crest. Hip circumference was measured at the widest point of buttocks, parallel to the ground. The blood plasma levels of total cholesterol (TC), HDL-cholesterol and triglycerides (TG) were marked with commercially available tests (Cormay, Poland). Serum insulin concentration was measured with the radioimmunological test (BioSource Europe S.A., Belgium), and the glucose level was determined with an enzymatic method (Cormay, Poland). Furthermore, all subjects underwent a standard two-hour Oral Glucose Tolerance Test (OGTT). A standard load of 75 g of glucose (Prolab, Poland) dissolved in 250 ml of distilled water was administered over 5 min. Venous blood was taken directly before (fasting state) and 120 min after glucose administration.

The LDL-cholesterol level was estimated with the Friedewald equation (Friedewald et al., 1972), and the Homeostatic Model Assessment of Insulin Resistance Index (HOMA_IR_) index was calculated using Matthews’ equation (Matthews et al., 1985).

### Training program

The training program consisted of aerobic, resistance exercises in the water to the accompaniment of music. The participants performed free exercises and exercises with props (flotation belts, rubber springs, dumbbell). The classes took place twice a week, and each class lasted 60 min. Each class began with a 10–12-min warm-up, which consisted of exercises of progressive intensity. Then, the participants performed aerobic exercises for 40 min, strength exercises for 5–10 min, and cool-down exercises for 2–3 min. The intensity of the aerobic exercises corresponded to 65–75% HR_max_ [HR_max_= 220 – age (years)]. The heart rate was measured with a Sport-Tester (Polar Elektro Oy, Finland). In the last 8–10 min of each session the participants performed stretching and relaxing exercises.

### Statistical analysis

The variables were presented using descriptive statistics: arithmetic means (*x̄*), standard deviations (SD), minimum (min) and maximum (max) values and confidence interval of 95% (95% CI). For variables with a normal distribution significance of differences between the measurements were assessed using the t-test. For variables with a non-normal distribution the non-parametric Wilcoxon signed-rank test was used. The level of statistical significance was set at p≤0.05. All statistical calculations were made using the STATISITICA 10 software package.

## Results

Table 1 shows the mean values (*x̄* ±SD) of somatic traits of the study participants, while Table 2 presents variables of lipid and carbohydrate metabolism measured before and after the training program. After the completion of the program, a significant reduction of the mean values of all somatic characteristics, i.e. body mass, BMI, waist circumference, hip circumference and the WHR index (p≤0.01) was registered. The highest statistically significant reduction of lipid profile components was found for total cholesterol, LDL-cholesterol and HOMA_IR_ (p≤0.01). The concentrations of triglycerides, insulin and glucose were reduced at p<0.05 (Table 2).

**Figure 1:**
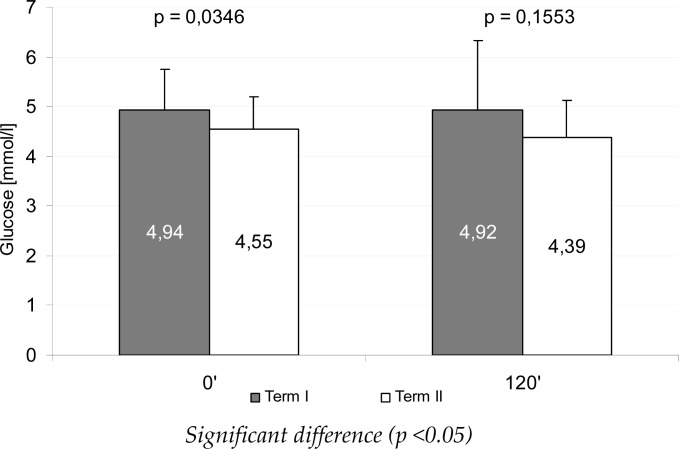
Mean glucose concentration during an oral glucose tolerance test (OGTT) between the measurements

**Figure 2:**
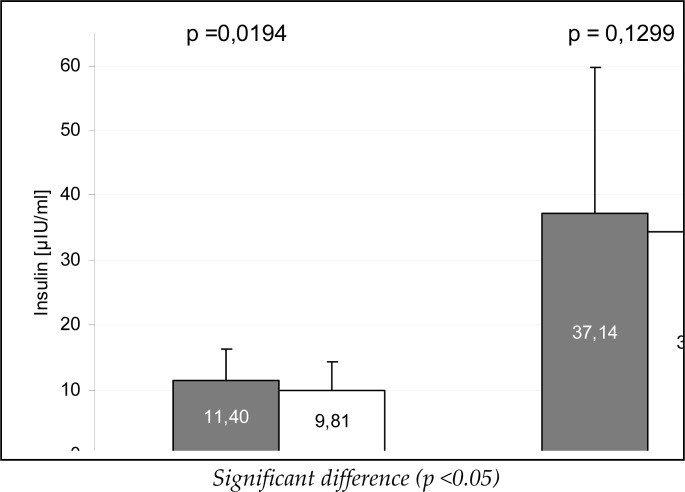
Mean insulin concentrations during an oral glucose tolerance test (OGTT) between the measurements

**Figure 3:**
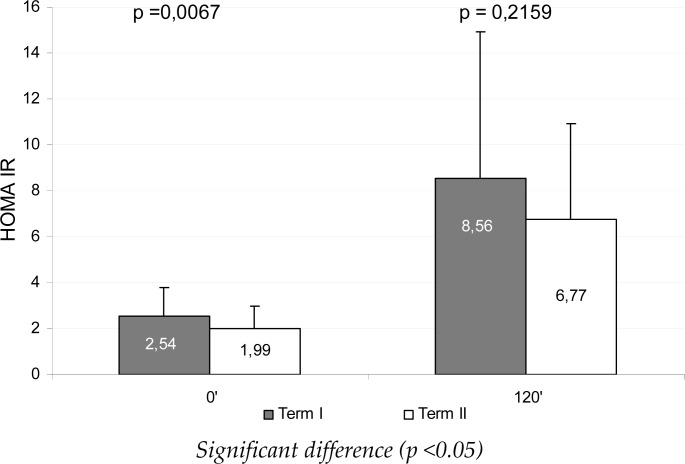
Mean HOMA_IR_ index during the oral glucose tolerance test (OGTT) between the measurements

Figures 1 and 2 present mean glucose and insulin levels during the OGTT test, during the measurements, whereas Figure 3 illustrates the HOMA_IR_ mean values. Between the measurements a significant reduction of fasting insulin, glucose and HOMA_IR_ was found (p<0.05), whereas after 120 min of the test the differences were statistically non-significant.

## Discussion

The obtained results correspond to those of other researchers indicating that systematic physical activity has a beneficial impact on morphological variables (Blair et al., 2001) and the blood lipid profile (Horowitz, 2001; Nowak et al., 2008) in obese individuals. These changes can be explained, on one hand, by the increased oxidation of free fatty acids and the improved function of the mitochondria (Horowitz, 2001), and on the other hand by increased insulin sensitivity (Pilaczyńska-Szcześniak et al., 2005; Mayer-Davis et al., 1998) due to the greater activity of GLUT-4 glucose transporters sensitive to muscle contraction (Cortright and Dohm, 1997; Thorell et al., 1999). It is emphasized that the intensivity of lipid metabolism during physical exercise in the water is conditioned by its temperature. During the thermoneutral immersion (water temperature 32–34 ° C) there is no heat loss from the human body. The main stimulus that increases the mobilization and oxidation of free fatty acids during prolonged exercise in such conditions is the activation of the sympathetic and adrenal-cortical system and increased release of catecholamines (Stokes et al., 2004). However, in lower water temperature not only the vasoconstriction of the skin occurs, also in the subcutaneous fat issue, which results in reduced blood flow to the tissues in which the fat is deposited. For this reason, mobilization and oxidation of free fatty acids is lower than that indicated by activation of the sympathetic nervous system (Wilmore and Costill, 2008).

The majority of studies on the effects of physical activity on lipid metabolism in individuals with abdominal obesity points to the normalization of HDL-cholesterol and TG levels, as the latter are considered to be the main source of free fatty acids utilized during physical activity (Horowitz, 2001). Regular physical exercise contributes to an increase of insulin-dependent lipoprotein lipase (LPL) in the adipose tissue and muscles as well as to the decreased activity of hepatic lipase, all of which leads to a reduction of the triglycerides level. An increase in the LDL-cholesterol level is related to the greater demand for free fatty acids as an energy substrate during muscle work and to the replenishment of muscle and cell membrane phospholipid supplies to repair eccentric exercise-induced myofibril damage (Nikilaidis et al., 2008). The restoration of intramuscular and membrane phospholipids by the fatty acids is thought to play the main role in the process of TG reduction in blood serum (Nikilaidis et al., 2008).

The implemented training program contributed to significant changes in the LDL-cholesterol level, which could have been related to the reduction of body girths and thus changes in body composition (Table 1). After the completion of the program BMI was lowered by 1.5%, and WHR by 4%. The noted reduction of total cholesterol (p<0.01) corresponds with the results of Thompson et al. (1979), according to whom an increased energy expenditure during physical exercise is conducive not only to a lower total cholesterol but may also induce a reduction of HDL-cholesterol level due to a decrease in the synthesis of total cholesterol as indicated by the results of the present study (Table 2).

The obtained results are contradictory to those of Viljoen and Christe (2011), who after a 24-week resistance training program (three times a week) in menopausal women, not only failed to find any positive changes in blood lipid profile, but also noted higher TG and LDL concentrations. In their study, Viljoen and Christe assumed no changes in the subjects’ body mass, while in the present work this assumption was not taken into account. Moreover, our training program, apart from resistance exercises, included also endurance exercises. Therefore, the observed positive changes in the blood lipid profile could be the effect of the beneficial impact of physical activity not only on body mass but, first and foremost, on body composition as confirmed by the WHR and BMI values. Our results correspond to earlier results of King et al. (1995) and Spate-Douglas and Keyser (1999).

The literature on the subject shows that regular physical exercise improves insulin sensitivity by increasing muscle contraction-related glucose transport (Santos et al., 2008; Farese, 2002; Fuerger et al., 2004), muscle blood flow (Suh et al., 2007) as well as expression and translocation of GLUT-4 glucose transporters and the encoding mRNA (McGee and Hargreaves, 2006). Physical exercise also increases the activity of phosphatidylinositide 3-kinase (PI3K) in the insulin receptor and the activity of insulin receptor substrate 1 (Arias et al., 2001; McGee and Hargreaves, 2006), thus increasing the receptor’s affinity to the agonist (Szcześniak et al., 1998). As far as carbohydrate metabolism is concerned a significant decrease of fasting insulin and glucose levels (p<0.05) and HOMA_IR_ (p<0.01) was noted after the completion of the training program. In the 120^th^ min of the OGTT test, the lower mean values of insulin and glucose concentrations were statistically non-significant. Pratley et al. (2000), after their training program lasting a few months (45–60 min, 3–4 times a week), noted a reduction in waist circumference, abdominal fat mass and insulin response in the OGTT, despite no changes in fasting glucose and insulin. The analysis of regression in Pratley et al. (2000) showed that changes in the waist circumference and percent of abdominal fat were independent factors of reduction of the late-phase insulin response during the OGTT. According to the American Heart Association and the American College of Sport Medicine, physical exercises of moderate intensity five times a week are recommended in preventive medicine (Haskell et al., 2007; Garber et al., 2011). Following these recommendations a concurrent decrease in the insulin and glucose levels should be obtained in the 120^th^ min of the test.

In the present study a 2.5 higher HOMA_IR_ in the first measurement (Table 2) was conditioned by both abdominal fat mass (Kahn and Flier, 2000) and age (Mazumdar et al., 2004). According to Ryan (2000), the most frequent cause for increasing insulin resistance with age is not age itself but the reduction of lean body mass, increasing visceral fat mass, neuro-hormonal changes and a lower physical activity level. The lower mean HOMA_IR_ found in the present study, after three months of systematic physical exercise in the water, allows to recognize the significant effects of physical activity on the development of peripheral insulin resistance.

## Conclusion

The obtained study results show that a 3 month aqua aerobics training program greatly contributed to positive changes in lipid metabolism, anthropometric variables and fasting levels of insulin, glucose and insulin resistance index. The participation in the training program did not, however, bring any changes in the resting HDL level and insulin, glucose and HOMA_IR_ levels in the 120^th^ min of the OGTT. Possibly greater weekly training loads or a longer training program might bring such changes.

## Figures and Tables

**Table 1:** Comparative analysis of somatic variables of studied subjects between the 1^st^ and 2^nd^ measurements

Variables	*x̄* ± SD (min ; max)		95% CI	p
	1^st^ measurement	2^nd^ measurement		
Body mass [kg]	87.08 ± 14.61 (65.8 ; 118.5)	85.73 ± 14.95 (63.5 ; 118.0)	0.71 – 1.99	0.0001
BMI [kg/m^2^]	32.60 ± 4.81 (25.7 ; 42.0)	32.11 ± 4.98 (24.2 ; 41.8)	0.27 – 0.74	0.0001
Waist circumference [cm]	101.63 ± 11.25 (80,0 ; 124,1)	95.34 ± 11.27 (77,0 ; 119,0)	4.90 – 7.67	< 0.0001
Hip circumference [cm]	115.41 ± 10.55 (96,0 ; 144,2)	111.00 ± 10.10 (92,3 ; 130,0)	2.20 – 6.62	< 0.0001
WHR	0.89 ± 0.05 (0.8 ; 1.0)	0.86 ± 0.05 (0.7 ; 1.0)	0.02 – 0.75	0.0002

Significant difference (p <0.05)

**Table 2:** Mean values (x ± SD) of variables of carbohydrate and lipid metabolism in studied subjects

Variables	*x̄* ± SD (min ; max)		95% CI	p
	1^st^ measurement	2^nd^ measurement		
TG [mg/dl]	99.79 ± 44.78 (38.8 ; 215.5)	91.66 ± 39.26 (41,0 ; 197.3)	2.36 – 13.91	0.0261
LDL [mg/dl]	111.33 ± 33.33 (53.3 ; 207.1)	102.03 ± 29.53 (56.3 ; 175.9)	3.65 – 14.95	0.0021
HDL [mg/dl]	59.54 ± 15.912 (33.2 ; 91.6)	58.37 ± 12.69 (37.8 ; 88.7)	0.01 – 4.18	0.3126
Total cholesterol [mg/dl]	190.81 ± 33.41 (133.8 ; 285.6)	179.36 ± 28.85 (130,0 ; 253.7)	5.80 – 17.10	0.0003
Glucose [mmol/l]	4.94 ± 0.80 (3.6 ; 7.0)	4.55 ± 0.63 (3.4 ; 6.4)	0.02 – 0.75	0.0377
Insulin [μIU/ml]	11.4 ± 4.842 (5.1 ; 22.3)	9.81 ± 4.47 (5,0 ; 25.7)	0.39 – 2.78	0.0194
HOMA_IR_	2.54 ± 1.24 (1.0 ; 5.5)	1.99 ± 0.97 (0.8 ; 5.5)	0.21 – 0.89	0.0067

Significant difference (p <0.05)
